# Cortical-spreading depression: at the razor’s edge of scientific logic

**DOI:** 10.1007/s10194-010-0287-z

**Published:** 2011-01-11

**Authors:** Vinod Kumar Gupta

**Affiliations:** Migraine-Headache Institute, S-407, Greater Kailash-II, New Delhi, 110 048 India

For over 60 years, cortical spreading depression (CSD) has mesmerized neurologists immersed in headache research. While science and logic are (or, at least, should be) inseparable, CSD—a largely experimental physiological reality—has dissociated the two in headache-related research. Although the need for a fresh pathophysiologic approach to migraine and other primary headaches unrelated to the neuronal (neural) or vascular theories has been voiced two decades ago [1, 2], and the issue has been questioned again recently [3, 4], the fascination for CSD as the basis for migraine remains largely undimmed. Currently, the pathogenetic role of CSD in migraine is widely accepted as an immutable fact or truth, and, any challenge to its role is regarded as almost heretical.

In this issue of TJHP, Yu and colleagues present an interesting facet of the mechanism of action of flunarizine in a rat model of CSD. The key finding is that flunarizine can alleviate cerebral mitochondrial injury under both normal and hypoxic conditions, a not unexpected effect attributed likely to blockade of voltage-gated calcium channels. However, hypoxia attenuated the protective effect of flunarizine on the CSD amplitude. In a nutshell, CSD leads to oxidative stress, or aggravated hypoxic conditions in the brain, and, these changes can be attenuated by flunarizine.

In extrapolating these experimental results in rats to humans, and, in particular, to migraine pathophysiology, certain features need careful consideration. The role of flunarizine in migraine therapeutics is itself uncertain. While flunarizine is used (albeit, with low-grade evidence) for migraine prophylaxis in several countries, it has yet to be approved for this indication by the United States Food and Drug Administration. Whereas the US FDA looks for evidences from randomized clinical trials (RCT) to stamp its approval of drugs such as flunarizine, the RCT itself is not without flaws, and, its utility in migraine is limited by the very nature of the disorder [5]. At a more fundamental level, flunarizine is a potent vasodilator of the cerebral vessels. Alcohol and nitroglycerin are well-established vasodilators that precipitate migraine predictably without any known effect(s) on CSD. There is, however, a significant clinical difference between sustained prophylactic cerebral vasodilatation (flunarizine) and relatively sudden cerebral vasodilatation (alcohol, nitroglycerin). Nevertheless, evidence that flunarizine can provide extended, satisfactory prophylaxis for most migraine patients is conspicuously sparse.

The limitations of CSD as a pathogenetic model for human neurological illnesses, particularly migraine, have been discussed almost exhaustively; additionally, a large and growing body of evidence indicates that CSD is biologically adaptive or neuroprotective [4]. There is not a single description of homonymously distributed migrainous scintillating scotoma; the scotoma has always been described as spreading towards the temporal field, right or left. Even Lashley [6] did not describe spread of his own migrainous scotoma towards the nasal field. As a neuropathologic concept, CSD has spawned much confusion and speculation, and, has outlived its usefulness. The time is, perhaps, ripe to bid farewell to an enduring and revered notion in neurology. Only then, we might step out of the maze of clinical and therapeutic assumptions that sustain a prominent role for CSD in human neuropathology. Such comprehension would also mark the beginning of the end of the stalemate that has gripped migraine research for over half a century.
